# Supplementary material for: Premature mortality and years of potential life lost from cardiovascular diseases: Protocol of a systematic review and meta-analysis
Source: PLoS One. 2023 May 3;18(5):e0284052. doi: 10.1371/journal.pone.0284052 (PMC10155956; doi:10.1371/journal.pone.0284052)
Supplement: S1 Table — (DOCX) [file pone.0284052.s002.docx]

**Table 1: Proposed Search Terms**

| Databases | Search terms |
| --- | --- |
| Web of Science | AB=("cardiovascular disease*" OR "CVD" OR "coronary disease*" OR "coronary heart disease*" OR "heart disease*" OR "cardiac disease*" OR "cardiac disorder*" OR "heart disorder*" OR "cardiac arrhythmia*" OR "cardiac dysrhythmia*" OR "atrial fibrillation*" OR "coronary artery disease*" OR "coronary arteriosclerosis" OR "Coronary Atherosclerosis" OR "myocardial ischemia*" OR "ischemic heart disease*" OR "myocardial infarction*" OR "cardiovascular stroke*" OR "heart attack*" OR "cardiogenic shock" OR "acute coronary syndrome*" OR "angina pectori*" OR "Cerebrovascular Disorders" OR "Cerebrovascular Disorders" OR "intracranial vascular disease*" OR "intracranial vascular disorder*" OR "cerebrovascular disease*" OR "brain vascular disorder*" OR "cerebrovascular occlusion*" OR "cerebrovascular insufficiency*" OR "brain ischemia" OR "carotid artery disease*" OR "cerebral vessel diseases" OR "intracranial arterial diseases" OR "intracranial hemorrhage*"OR "cerebral hemorrhage*" OR "stroke*" OR "cerebrovascular accident*" OR "CVA" OR "brain vascular accident*" OR "intracranial arteriosclerosis" OR "cerebral arteriosclerosis" OR "cerebral atherosclerosis" )  AND  AB=("behavioral risk*" OR "behavioral factor*" OR "modifiable risk*" OR "non communicable disease*" OR “NCD” OR "Hypertension" OR "blood pressure" OR "Obesity" OR "obese" OR "overweight" OR "Body Weight" OR "body mass index" OR "hyperglycemia*" OR "blood glucose" OR "diabetes” OR "DM" OR "diabetic" OR "blood sugar" OR "hyperlipidemia*" OR "hypercholesterolemia*" OR "cholesterol*" OR "Hypercholesteremia" OR "low density lipoprotein" OR "high density lipoprotein" OR "triglyceride*" OR "triacylglycerol*" OR “Metabolic Risk Factor*” OR Tobacco OR smoking OR "smoker*" OR "Nicotine" OR "cigarette*" OR "E-Cigarette" OR "electronic cigarette*" OR "vape*" OR "ecig" OR “Sedentary" OR "physical inactivity" OR " physical activity" OR "alcohol" OR "Alcoholism" OR "binge drinking" OR "drinking" OR "diet" OR "poor nutrition" OR “sugar” OR "sweetened beverage*" OR "sweetened drink*" OR "sweet*" OR "manufacture drink*" OR "fat*" OR "Food*" OR "low fiber" OR "socioeconomic*" OR "inequality*" OR "low income" OR "low education" OR "poverty" OR "indigent*" OR "disparities" OR sex OR gender OR “male*” OR “female*”)  AND  TI=("premature mortality" OR "premature death" OR "years of life lost" OR "YLL" OR "Potential Years of Life Lost" OR "PYLL" OR "life expectancy " ) |
| Pubmed | Search: cardiovascular disease[MeSH Terms]  AND  Search: "behavioral risk*"[Title/Abstract] OR "behavioral factor*"[Title/Abstract] OR "modifiable risk*"[Title/Abstract] OR "non communicable disease*"[Title/Abstract] OR "NCD"[Title/Abstract] OR "Hypertension"[Title/Abstract] OR "blood pressure"[Title/Abstract] OR "Obesity"[Title/Abstract] OR "obese"[Title/Abstract] OR "overweight"[Title/Abstract] OR "Body Weight"[Title/Abstract] OR "body mass index"[Title/Abstract] OR "hyperglycemia*"[Title/Abstract] OR "blood glucose"[Title/Abstract] OR "diabetes"[Title/Abstract] OR "DM"[Title/Abstract] OR "diabetic"[Title/Abstract] OR "blood sugar"[Title/Abstract] OR "hyperlipidemia*"[Title/Abstract] OR "hypercholesterolemia*"[Title/Abstract] OR "cholesterol*"[Title/Abstract] OR "Hypercholesteremia"[Title/Abstract] OR "low density lipoprotein"[Title/Abstract] OR "high density lipoprotein"[Title/Abstract] OR "triglyceride*"[Title/Abstract] OR "triacylglycerol*"[Title/Abstract] OR "Metabolic Risk Factor*"[Title/Abstract] OR tobacco[Title/Abstract] OR smoking[Title/Abstract] OR "smoker*"[Title/Abstract] OR "Nicotine"[Title/Abstract] OR "cigarette*"[Title/Abstract] OR "E-Cigarette"[Title/Abstract] OR "electronic cigarette*"[Title/Abstract] OR "vape*"[Title/Abstract] OR "ecig"[Title/Abstract] OR "Sedentary"[Title/Abstract] OR "physical inactivity"[Title/Abstract] OR " physical activity"[Title/Abstract] OR "alcohol"[Title/Abstract] OR "Alcoholism"[Title/Abstract] OR "binge drinking"[Title/Abstract] OR "drinking"[Title/Abstract] OR "diet"[Title/Abstract] OR "poor nutrition"[Title/Abstract] OR "sugar"[Title/Abstract] OR "sweetened beverage*"[Title/Abstract] OR "sweetened drink*"[Title/Abstract] OR "sweet*"[Title/Abstract] OR "fat*"[Title/Abstract] OR "Food*"[Title/Abstract] OR "low fiber"[Title/Abstract] OR "socioeconomic*"[Title/Abstract] OR "inequality*"[Title/Abstract] OR "low income"[Title/Abstract] OR "low education"[Title/Abstract] OR "poverty"[Title/Abstract] OR "indigent*"[Title/Abstract] OR "disparities"[Title/Abstract] OR sex[Title/Abstract] OR gender[Title/Abstract] OR "male*"[Title/Abstract] OR "female*"[Title/Abstract]  AND  Search: "premature mortality"[Title] OR "premature death"[Title] OR "years of life lost"[Title] OR "YLL"[Title] OR "Potential Years of Life Lost"[Title] OR "PYLL"[Title] OR "life expectancy loss" [Title] OR "standardized mortality ratios"[Title] OR "standardized mortality rate*"[Title] OR "Life Expectancy"[Title] |
| Scopus | TITLE-ABS-KEY ( "cardiovascular disease*" OR "CVD" OR "coronary disease*" OR "coronary heart disease*" OR "heart disease*" OR "cardiac disease*" OR "cardiac disorder*" OR "heart disorder*" OR "cardiac arrhythmia*" OR "cardiac dysrhythmia*" OR "atrial fibrillation*" OR "coronary artery disease*" OR "coronary arteriosclerosis" OR "Coronary Atherosclerosis" OR "myocardial ischemia*" OR "ischemic heart disease*" OR "myocardial infarction*" OR "cardiovascular stroke*" OR "heart attack*" OR "cardiogenic shock" OR "acute coronary syndrome*" OR "angina pectori*" OR "Cerebrovascular Disorders" OR "Cerebrovascular Disorders" OR "intracranial vascular disease*" OR "intracranial vascular disorder*" OR "cerebrovascular disease*" OR "brain vascular disorder*" OR "cerebrovascular occlusion*" OR "cerebrovascular insufficiency*" OR "brain ischemia" OR "carotid artery disease*" OR "cerebral vessel diseases" OR "intracranial arterial diseases" OR "intracranial hemorrhage*" OR "cerebral hemorrhage*" OR "stroke*" OR "cerebrovascular accident*" OR "CVA" OR "brain vascular accident*" OR "intracranial arteriosclerosis" OR "cerebral arteriosclerosis" OR "cerebral atherosclerosis" )  AND  TITLE-ABS-KEY ( "behavioral risk*" OR "behavioral factor*" OR "modifiable risk*" OR "non communicable disease*" OR "NCD" OR "Hypertension" OR "blood pressure" OR "Obesity" OR "obese" OR "overweight" OR "Body Weight" OR "body mass index" OR "hyperglycemia*" OR "blood glucose" OR "diabetes" OR "DM" OR "diabetic" OR "blood sugar" OR "hyperlipidemia*" OR "hypercholesterolemia*" OR "cholesterol*" OR "Hypercholesteremia" OR "low density lipoprotein" OR "high density lipoprotein" OR "triglyceride*" OR "triacylglycerol*" OR "Metabolic Risk Factor*" OR tobacco OR smoking OR "smoker*" OR "Nicotine" OR "cigarette*" OR "E-Cigarette" OR "electronic cigarette*" OR "vape*" OR "ecig" OR "Sedentary" OR "physical inactivity" OR " physical activity" OR "alcohol" OR "Alcoholism" OR "binge drinking" OR "drinking" OR "diet" OR "poor nutrition" OR "sugar" OR "sweetened beverage*" OR "sweetened drink*" OR "sweet*" OR "manufacture drink*" OR "fat*" OR "Food*" OR "low fiber" OR "socioeconomic*" OR "inequality*" OR "low income" OR "low education" OR "poverty" OR "indigent*" OR "disparities" OR sex OR gender OR "male*" OR "female*" )  AND  TITLE ( "premature mortality" OR "premature death" OR "years of life lost" OR "YLL" OR "Potential Years of Life Lost" OR "PYLL" OR "life expectancy loss" ) |
| CENTRAL | MeSH descriptor: [Cardiovascular Diseases] explode all trees  AND  "premature mortality" OR "premature death" OR "years of life lost" OR "YLL" OR "Potential Years of Life Lost" OR "PYLL" OR "life expectancy loss" |
